# Skin avulsion injury during endotracheal tube extubation – case report of an unusual complication

**DOI:** 10.1186/1754-9493-2-12

**Published:** 2008-05-21

**Authors:** Berkhan Yilmaz, Kutay Colakoglu, Raffi Gurunluoglu

**Affiliations:** 1 Department of Plastic, Reconstructive & Aesthetic Surgery, Acibadem Hospital, Istanbul, Turkey; 2 Department of Anesthesiology, Acibadem Hospital, Istanbul, Turkey; 3 Plastic and Reconstructive Surgery, Denver Health Medical Center, University of Colorado Health Sciences, Denver, Colorado, USA

## Abstract

We report a geriatric case with a full-thickness skin avulsion injury during extubation due to a tube securing tape used to fixate the endotracheal tube. The avulsed skin was sutured back to its original place. Based on this single geriatric patient, we recommend anesthesiologists/anesthetists and surgeons be aware of the potential risk of avulsing the skin with tape during a standard extubation procedure. This may especially occur in geriatric patients who have age related changes as decreased elasticity and resistance to shearing forces that predispose the skin to get traumatized easily.

## Background

Endotracheal tubes that are used for airway maintenance must be securely placed and fixated in order to avoid complications such as accidental extubations, tracheal injuries or swallowed endotracheal tube [1].

We report a 75-year-old patient in whom a full-thickness skin avulsion injury occurred during extubation due to securing tape used to fixate the endotracheal tube. In addition, plausable reasons of this rare complication are discussed.

## Case presentation

**Figure 1:**
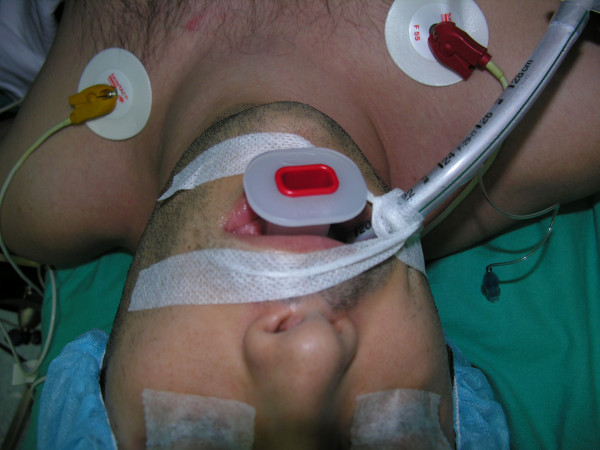
A case example of how the endotracheal tube is fixated in a standard patient (front view).

**Figure 2:**
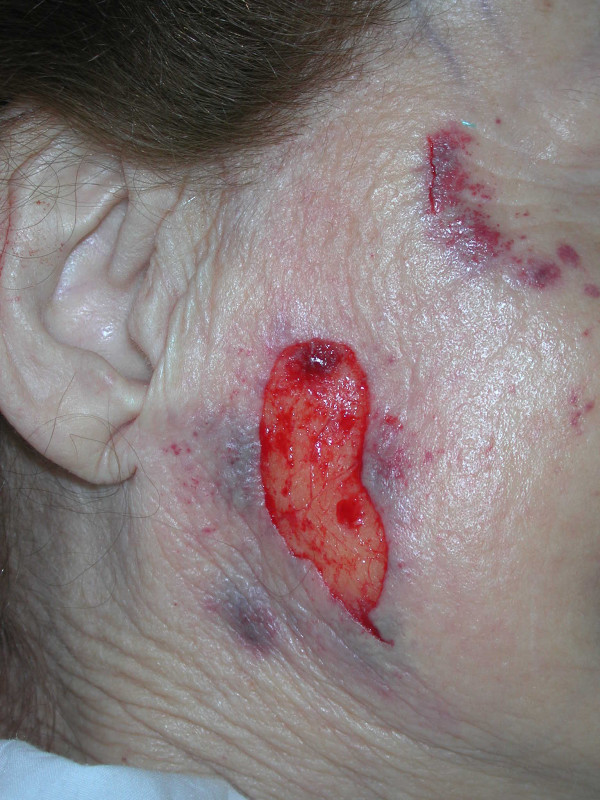
Skin flap avulsion during extubation due to adhesive tape.

**Figure 3:**
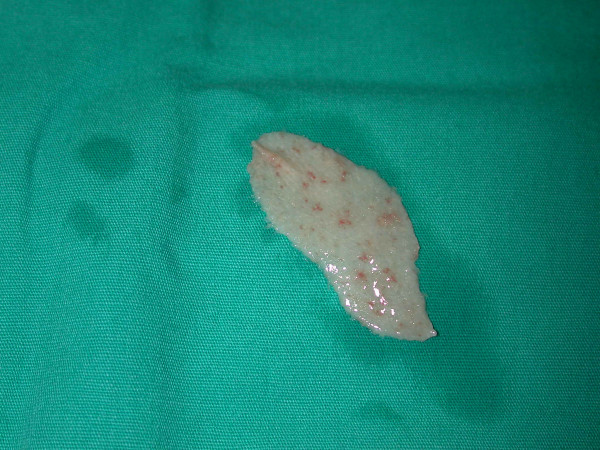
Defatting of the skin just before its placement as a skin graft.

**Figure 4:**
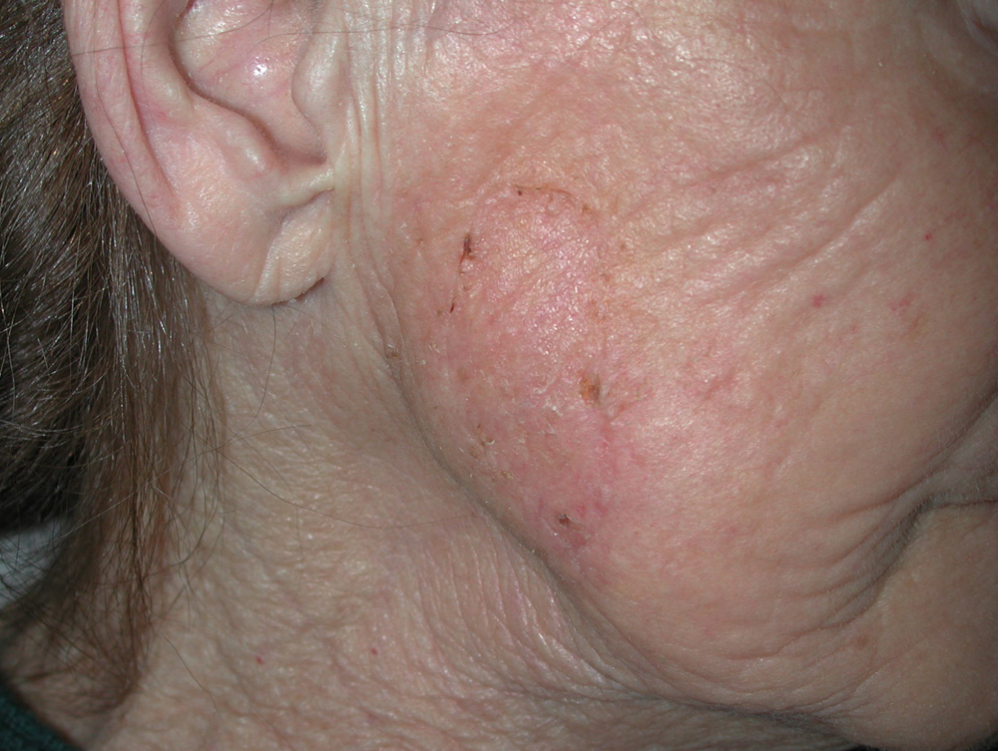
Full take of the skin graft following replacement as a graft (1 week after surgery).

A 75-year-old Caucasian female was admitted to the emergency department after falling down the stairs. On examination, there was a severe tenderness on her right hip and bruises on her both cheeks, on right malar region and dorsal region of her left forearm. No open wound was detected. Her skin turgor and tonus were seriously decreased. Her medical history was normal. Radiological studies showed a right hip fracture. A total hip joint replacement as an emergency procedure was planned by orthopedics. The patient was intubated by an anesthesiologist with an endotracheal tube and the tube was fixated and secured to the patient's right cheek by using tapes (Hypafix^®^;BSN medical GmgH & Co. KG, Hamburg, Germany) in a standard fashion [2] (Fig. 1). During a standard extubation, a piece of full-thickness skin avulsed as attached to the securing tape from the patient's right cheek. Plastic surgery department was urgently consulted. The skin graft was removed from the tape, and it was preserved in a gauze soaked into saline (Fig. 2). On examination, a full-thickness skin loss 3 × 8 cm in size was detected on the right cheek (Fig. 3). The avulsed skin was sutured back to its original place by 5/0 plain catgut sutures under sedation and local anesthesia. Being aware of the patient's fragile skin, the tie-over sutures used routinely to immobilize the graft were passed deep enough to avoid undue tears of the overlying epithelium. Seven days later after an uneventful healing period, the tie-over dressing was opened and the graft was found to heal completely (Fig. 4).

## Discussion

Airway control is the first step of patient care requiring either short term or long term respiratory support, regardless of the primary pathology. Proper endotracheal intubation and safe fixation of the tube to prevent tube dislodgement are two critical steps for airway maintenance [2-6].

Several articles discussed the tube fixation methods in neonates and pediatric cases, because of their delicate skin structure to avoid complications [1,3]. We believe special attention must also be paid in geriatric patients.

Cumulative effect of several factors seemed to have caused an avulsion skin injury in the presented case. One such factor was the aging of the skin. Aging is a process of loosing the properties of skin as well as resisting to the gravitational forces. At the histologic level, cutaneous aging is manifested by flattening of the dermal-epidermal junction, a decrease in the number of melanocytes and Langerhans cells, a reduction in the amount of glycosaminoglycan ground substance, progressive dropout of elastic fibers, and diminution in the total amount of collagen as well as the fraction of type III collagen. Superimposed on the inevitable changes outlined above are the effects of sun damage, which result in epidermal dysplasia and dermal elastosis, and dramatically exacerbate the deterioration inherent in the aging process. The histologic deterioration of the skin due to aging correlates with the clinical findings: thinning of the skin, decreased resistance to shearing forces, decreased elasticity [7].

Another important factor that may have contributed to this compliction was the presence of prior blunt injury to the right cheek. Additionally, the pulling force of the tape applied by the anesthesiologist also seemed to have played a significant role in the occurrence of this unfortunate injury.

## Conclusion

We recommend anesthesiologists/anesthetists and surgeons be aware of the potential risk of avulsing the skin with tape during a standard extubation procedure. This may especially occur in geriatric patients who have age related changes such as decreased elasticity and resistance to shearing forces that predispose the skin to get traumatized easily, especially when a preexisting blunt trauma is present. We believe this complication could have been avoided by securing the tape to a non-traumatized skin areas, by less forceful and careful pull of the tube and the tape.

## Authors' contributions

BY performed the skin grafting to the face following injury, BY wrote the manuscript, KC participated in the surgery as the anesthesiologist, RG participated in editing extensively and in the submission of the manuscript. All authors read and approved the final manuscript.

## Consent

A written informed consent was obtained from the patient for publication of this case report and any accompanying images. A copy of the written consent is available for review by the Editor-in-Chief of this journal.
